# Voltammetric lipase activity assay based on dilinolein and a modified carbon paste electrode

**DOI:** 10.1007/s00216-022-04135-y

**Published:** 2022-05-31

**Authors:** Anita Rogala, Julia Rechberger, Vanessa Vasold, Anchalee Samphao, Kurt Kalcher, Astrid Ortner

**Affiliations:** 1 Institute of Pharmaceutical Sciences, Department of Pharmaceutical Chemistry, University of Graz, Schubertstraße 1, 8010 Graz, Austria; 2 Department of Chemistry, Faculty of Science, Ubon Ratchathani University, Ubon Ratchathani, 34190 Thailand; 3 Institute of Chemistry, Department of Analytical Chemistry, University of Graz, Universitätsplatz 1, 8010 Graz, Austria

**Keywords:** Cobalt(II)phthalocyanine, Carbon nanotubes, Inhibition, Orlistat

## Abstract

In this work, a novel electrochemical assay for characterizing both lipases and lipase inhibitors as well as for the determination of lipase activity is described. It is based on a carbon paste electrode, modified with cobalt(II)phthalocyanine, and multi-walled carbon nanotubes (MWCNTs). As reaction media, a sodium borate buffer was used (0.1 M, pH 9). The measurements were carried out in a batch system using differential pulse voltammetry (DPV) and 1,3-dilinolein as standard substrate. The activity assay showed a linearity for porcine pancreas lipase activity in a range between 20 and 300 U L^−1^ (per min) with a limit of detection (LOD) of 7 U L^−1^ and a limit of quantification (LOQ) of 20 U L^−1^. The kinetic behavior of the lipase reaction was investigated, resulting in a *K*_M_ value of 0.29 mM. The applicability of the activity assay could be shown by investigating the activity of lipases from *Aspergillus oryzae* and *Candida rugosa*, and the results were confirmed by a reference method. The inhibitory effects were characterized with Orlistat.

**Graphical abstract:**

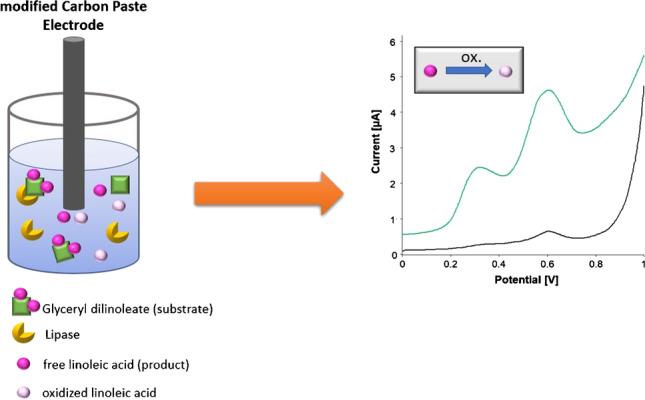

**Supplementary Information:**

The online version contains supplementary material available at 10.1007/s00216-022-04135-y.

## Introduction

Lipases are water-soluble, ubiquitous enzymes, found in most organisms, such as mammals, plants, bacteria, or molds [1, 2]. They are a subclass of esterases, primarily hydrolyzing the ester bonds of triglycerides, generating diglycerides and fatty acids. These diglycerides are further degraded to β-monoglycerides and fatty acids, which can then be easily transported to the enterocytes [1, 3]. Two things make the lipase unique among the enzymes: firstly, it is their ability to react at the interface between the aqueous and the oil phases. This ability is necessary due to the opposite polarity between the enzyme (hydrophilic) and the substrate (lipophilic) [1]. Secondly, it is their special structure. Most lipases have a lid region (an α-helical loop), which covers their active site. Substrates can only react with the active site when the lid is in the open state. The activity of the lipase, hence, depends on the conformational changes of the lid region [1, 3].

Most lipases are also regiospecific, preferring the ester bond at the Sn-1,3 position of the triglyceride. The generated monoglycerides are stable to further degradation, if their ester bond is at the Sn-2 position [1, 2]. Besides hydrolyzation, lipases are also a powerful catalyst for other reactions, such as esterification or transesterification. Since they can be produced in high quantities by microbial organisms, they are therefore widely used as detergent additives in cleaning agents, as well as in the pharmaceutical, food, or cosmetic industry. Furthermore, their application also extends to leather and paper industry [2, 4–6].

Hence, the lipase activity is of essential importance. Several methods for lipase activity determination have therefore been reported. Detailed reviews by Beisson et al. [7], Stoytcheva et al. [8], or Hasan et al. [9] showed an overview of the recent methods used for lipase activity measurement. Overall, they can be categorized into the following methods: volumetry, spectrometry, conductometry, immunoassays, radioactive assays, chromatography, or enzymatic assays (e.g., biosensors) [10]. An electrochemical detection of lipase activity was described by Valincius et al. [11] using 9-(5′-ferrocenylpentanoyloxy)-nonyl disulfide (FPONDS) on gold electrodes, modified by a hexanethiol self-assembled monolayer. The lipase hydrolyzes the ester bonds of FPONDS molecules, generating ferrocene groups of FPONDS, thus triggering an electrochemical redox signal. Ignatjev et al. [12] reported a direct amperometric determination method for lipase activity by using O-palmitoyl-2,3-dicyanohydroquinone (PDCHQ) oxidation as lipase substrate and measuring the increasing current, which was proportional to the amount of lipase added. Fuse et al. [4] developed a lipase assay via flow injection analysis with olive oil as substrate, measuring the reduction of 2-methyl-1,4-naphthoquinone (vitamin K_3_), which is proportional to the fatty acid concentration. The formed emulsion is injected into the FIA system. The flow signal was corresponding to the lipase activity. Commercially available digestive preparations were studied. An electrochemical determination of lipase enzyme activity was reported by Reddy et al. [13], investigating the lipase inhibition by organophosphorus pesticides, using p-nitrophenyl acetate as a substrate, which was hydrolyzed to p-nitrophenol by the lipase. The concentration of organophosphorus pesticides can be determined via the inhibition of lipase activity. Kumara et al. [14] reported the development of a polyaniline-coated carbon electrode for the detection of lipase. The latest approach was described by Zlateva et al. [15], who designed an impedimetric sensor for the quantification of lipase activity with olive oil as substrate.

In the modern world, fats (especially triglycerides) and sugars are a major source of energy in our diets. The inhibition of triglyceride uptake is considered an important therapy to decrease cardiovascular diseases and obesity. Orlistat, a synthetic derivate of lipstatin, is one of the most common lipase inhibitors. By bonding with the active serine site of the lipase, it inhibits the hydrolyzation of dietary fats (as triglycerides), thus leading to elimination of the undigested triglycerides via the fecal route [16–18]. Thus, the calorie intake of the patients is decreased. The characterization of inhibitor behavior is therefore an interesting aspect for the development of new medication.

Although there are already existing methods for lipase activity determination, quite a few of them have common disadvantages: either complicated or time-consuming pretreatments, temperature-dependent measurement conditions, or the usage of chemically not well-defined substrates such as olive oil. The aim of this work was therefore to develop a simple and time-saving electrochemical assay without pre-treatment of the electrode or the sample, for both the determination of lipase activity in solution and the characterization of the behavior of lipase inhibitors, which can be used at room temperature. As a measuring system, an electrochemical sensor was used, which was already described by this working group [19]. It consists of a carbon paste electrode (CPE), modified with cobalt(II)phthalocyanine (Co(II)PC), and multi-walled carbon nanotubes (MWCNTs). It is a new, easy, and inexpensive technique that does not require cost-intensive reagents or materials.

## Experimental

### Chemicals and materials

The porcine pancreas lipase (EC 3.1.1.3, type II, activity: 100–500 units/mg protein using olive oil, 30–90 units/mg protein using triacetin as substrates; protein content 40%), lipase from *Aspergillus oryzae* (EC 3.1.1.3, activity: ≥ 100,000 units/g protein using glycerol tributyrate as substrate), and lipase from *Candida rugosa* (EC 3.1.1.3, type VII, activity: ≥ 700 units/mg protein using olive oil as substrate) were obtained from Sigma-Aldrich Handels Gmbh (Vienna, Austria). The standard substrate dilinolein (1,3-dilinoleoyl-glycerol) was purchased at Santa Cruz Biotechnology. The other substrates monolinolein (glyceryl monolinoleate), trilinolein (glyceryl trilinoleate), ethyl linoleate, and linoleic acid, as well as cobalt(II) phthalocyanine, graphite (< 20 micron powder), multi-walled carbon nanotubes (O.D. × L: 110–170 nm × 5–9 µm; > 90% carbon), tris(hydroxymethyl)aminomethane and boric acid, were also purchased at Sigma-Aldrich Chemicals. Sodium hydroxide was ordered from Carl Roth GmbH & Co. KG (Karlsruhe, Germany). Paraffin oil, potassium dihydrogen phosphate, disodium hydrogen phosphate, and hydrochloric acid were obtained from Merck GesmbH (Vienna, Austria). All chemicals and reagents were of analytical grade and used without further purification.

The inhibitor Orlistat (Hexal) was used as its pharmaceutical preparation (Orlistat HEXAL®; 60 mg) and was bought at a local pharmacy.

### Apparatus and preparation of the working electrode

Differential pulse voltammetric (DPV) measurements were carried out with a Metrohm 797 VA Computrace with the 1.3 software. The electrochemical system consisted of a working electrode, a platinum auxiliary electrode, and a Ag/AgCl 3 M KCl reference electrode. The working electrode is a rodlike carbon paste electrode (length 51.0 mm, diameter of carbon paste area 3.0 mm).

The preparation of the working electrode was performed according to Jerkovic et al. [19] as follows: 12.5 mg Co(II)PC, 150 mg paraffin oil, 25.0 mg MWCNTs, and 310 mg graphite powder were blended to a homogenous paste. Before the application, the carbon paste was then put in the fridge overnight. For the voltammetric measurements, the paste was filled into a rodlike working electrode and polished on a Teflon disk to ensure a smooth surface. Freshly prepared electrodes can be stored at least 2 weeks in the fridge without loss of activity.

### Measurement conditions

The DPV measurements were carried out in a batch system by applying the following conditions: potential ramp 0.0 to 1.0 V; pulse amplitude 0.05 V; pulse time 0.04 s; voltage step 0.002 V; voltage step time 0.3 s, and sweep rate 0.0066 V s^−1^.

All measurements were carried out in aqueous buffer solutions. Sodium borate/NaOH buffer (0.1 M, pH 9) was prepared by dissolving boric acid and sodium hydroxide and adjusting the pH with NaOH 0.1 M. Tris/HCl buffer (0.2 M, pH 9) was made by dissolving tris(hydroxymethyl)aminomethane and adding HCl 0.1 M until the requested pH is achieved. Sorensen phosphate buffer (0.1 M, pH 9) was manufactured by mixing a 0.1 M Na_2_HPO_4_* 2 H_2_O solution and 0.1 M KH_2_PO_4_ solution until the respective pH is gained. For all preparations, high-purity deionized water was used.

For the basic investigations, the porcine pancreas lipase (PP-L) powder was used. A stock solution was prepared by mixing 10 mg of the PP-L powder in 1 mL water. The solution was then centrifuged at 4 °C for 2 min with 3000 rpm; the supernatant was used for the measurements. Amounts of 0.5–38 mg of lipases from *Candida rugosa* (CR–L) and *Aspergillus oryzae* (AO-L) were mixed in 1 mL water. Again, the stock solutions were centrifuged, and the supernatant was used for measurements.

The used supernatants of the stock solutions will be referred as lipase solution (LS) in the following text, and if a specific lipase is described, it will be referred to as PP-LS, CR-LS, or AO-LS.

The substrates (monolinolein, dilinolein, and trilinolein) were each dissolved in paraffin oil. The measuring cell was filled with 5 mL buffer solution. After determining the blank value, 900 μL LS was added, followed by 100 μL of the respective substrate solution (5–45 mM). The measurement was carried out after a reaction time of 0–30 min. Peaks were evaluated via tangent method. It is necessary to use new electrodes for each measurement.

The inhibitor solution was prepared by dissolving the respective amount of Orlistat in 1 mL ethanol 96%.

### Determination of kinetic parameters

The kinetic parameters, *K*_M_ and *I*_max_, were determined as described above by using 900 μL PP-LS and 100 μL dilinolein (7 mg mL^−1^, 14 mg mL^−1^, and 28 mg mL^−1^ in paraffin oil) as substrate and measuring the reaction from 0 to 15 min at room temperature in a sodium borate/NaOH buffer (0.1 M, pH 9). The lower the *K*_M_, the higher the affinity of the lipase towards the substrate.

### Determination of inhibitory effects

For the determination of the inhibitory effect, first the blank value was determined, followed by addition of 900 μL PP-LS and 10 μL Orlistat in ethanol 96% solution (0.03–0.12 mg mL^−1^). Subsequently, 100 μL substrate solution (10 mM) was injected into the measurement cell. The measurement was carried out after a reaction time of 0–30 min.

### Titrimetric method

As reference method, a titration was performed. The method was provided by the lipase supplier (Sigma-Aldrich), based on the work of Sullivan and Howe [20], using triacetin as substrate. The first step was to prepare a buffered triacetin solution (150 mM Tris HCl buffer with 330 mM triacetin with a pH 7.4 at 37 °C). The triacetin buffer was then mixed with a lipase solution (150–300 U mL^−1^ of lipase (PP-L, CR–L, and AO-L) in cold deionized water) in a ratio of 3:1 (v:v) and incubated for 60 min at 37 °C under constant stirring. Subsequently, 4 drops of thymolphthalein were added. The titration was carried out immediately using NaOH 0.1 M to a pale blue endpoint.

One unit lipase will hydrolyze 1.0 microequivalent of fatty acid from a triglyceride in 1 h at pH 7.4 at 37 °C.

### Biuret reaction

The Biuret reagent was prepared by mixing potassium sodium tartrate tetrahydrate (32 mM), potassium iodide (18 mM), copper(II) sulfate (12 mM), and NaOH (200 mM). A calibration curve was made by dissolving bovine serum albumin in Milli-Q water (0.0025–0.01 g mL^−1^). The lipases (PP-L, CR–L, and AO-L) were also dissolved in high-purity deionized water (0.01–0.20 g mL^−1^). For the photometric determination, 2.5 mL of the Biuret reagent was mixed with 50 μL of the lipase solution. Biuret reagent was used as blank. After 30-min reaction time, the absorption was measured at 545 nm.

## Results and discussion

### Development of the activity assay

**Fig. 1:**
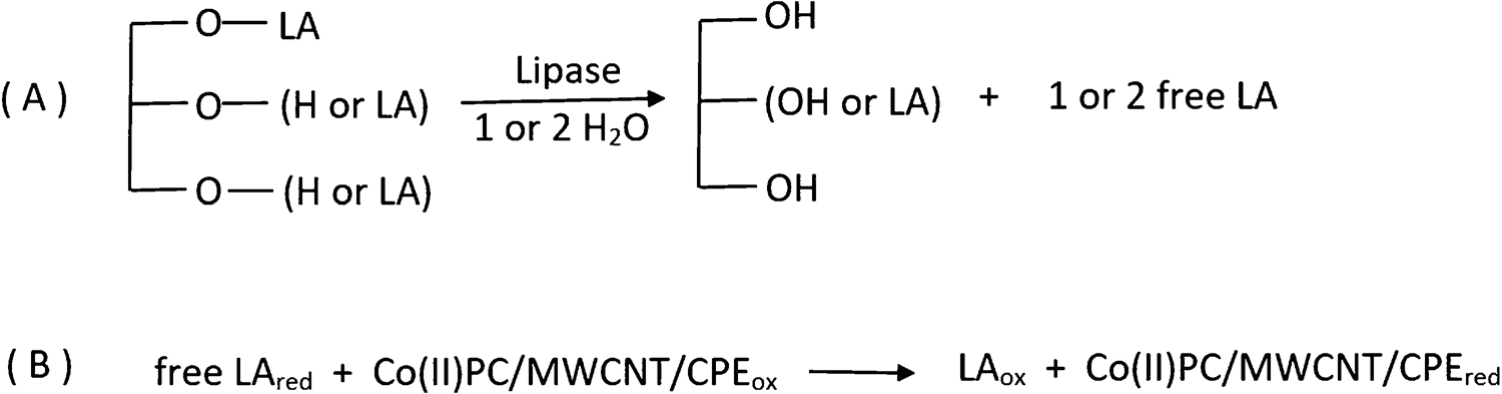
(A) Reaction principle of a glyceryl linoleates in the presence of lipase and water. Glycerol and linoleic acid are formed. (B) The linoleic acid is being oxidized at the Co(II)PC/MWCNT-modified CPE

The assay is based on the enzymatic reaction of a specific substrate glyceryl (mono-, di-, or tri-) linoleate in the presence of lipase and water. Linoleic acid (LA) is formed as product, which can be detected at the Co(II)PC/MWCNT-modified electrode. The general principle of the assay is shown in Fig. 1.

To test the principle of the activity assay, DPV measurements at the Co(II)PC/MWCNT-modified CPE with different substrates in the presence of lipase were carried out.

The active surface area was estimated by a comparison of current densities of the modified electrode with a quasi-planar glassy carbon electrode using hexacyanoferrate as a probe under the given experimental conditions (sodium borate buffer 0.1 M, pH 9). It was found that it is approximately 1.8 times the geometric area, due to the high roughness of the carbon paste surface. Furthermore, cyclic voltammetric (CV) measurements, also with hexacyanoferrate as a probe, were carried out under the same conditions by applying different scan rates (0.01–0.5 V s^−1^), showing a linear dependence of the peak current and the square root of the scan rate (see Supplementary Information Fig. S1). EIS measurements were performed for the used carbon paste electrode in a previous work, demonstrating an improved charge transfer resistivity of the carbon paste by MWCNTs and a decrease after the addition of cobalt(II)phthalocyanine to the carbon paste. Nevertheless, the measurement in DPV showed increased peaks when cobalt(II)phthalocyanine and MWCNTs were added in the carbon paste. It was assumed that this is due to the adsorption of the linoleic acid via the MWCNTs [19].

**Fig. 2:**
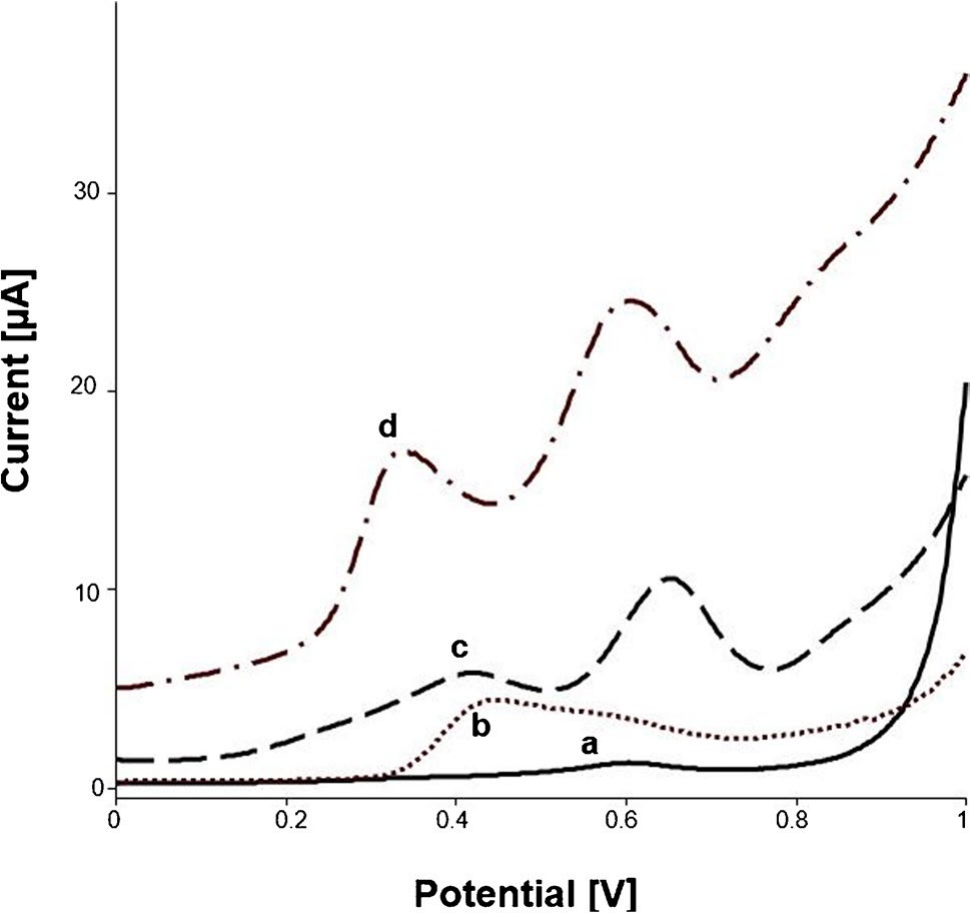
Typical voltammogram of (a) blank. (b) Addition of 900 µL PP-LS (10 mg mL^−^^1^). (c) After injection of 100 µL dilinolein (10 mM). (d) Injection of 200 µL linoleic acid (10 mM) as comparison. All measurements were performed in sodium borate buffer (0.1 M, pH 9)

As could already be demonstrated by this working group, linoleic acid shows two well-defined peaks at a potential of around + 0.35 V and + 0.5 V [19] (see Fig. 2, curve d).

When adding PP-LS to the buffer solution, a small peak at + 0.5 V was obtained (see Fig. 2, curve a). After addition of glyceryl linoleate to the PP-L containing measuring cell, two peaks at + 0.4 V and + 0.65 V were formed, after a reaction time of 25 min (see Fig. 2, curve c). For the evaluation of the lipase activity, the first peak (oxidation of linoleic acid) was used because the second one (oxidation of Co(II)PC) exhibited lower selectivity due to the higher potential.

Hence, the setup of the electrochemical activity assay was confirmed.

### Optimization of the activity assay

For the initial experiments, PP-L was used as lipase and trilinolein acted as substrate. Since it contains three fatty acid groups, it was expected to be a promising reaction partner.

For the optimization of the measurement system, the first step was the determination of the buffer system and the reaction time. Therefore, three well-known buffers were used: Sorensen phosphate (0.1 M), Tris HCl (0.1 M), and sodium borate buffer (0.1 M) in a pH range of 7–9 with varying reaction times from 0 to 30 min. The investigations showed that there was no signal at a pH < 6 but with increasing peaks with higher pH values. The best results concerning peak height and precision of the method were obtained with sodium borate buffer (0.1 M) at pH 9 after 25 min, resulting in a peak increase of 54% compared to Sorensen phosphate and 75% compared to Tris HCl. The found optimum at pH 9 is also in accordance to literature, stating pH optima at slightly alkaline pH values occur due to the natural reaction of lipase in the duodenum [1, 21].

After determining the best buffer system, the reaction temperature was investigated. Three temperatures were tested: 10 ± 2 °C, 22 ± 2 °C (room temperature), and 35 ± 2 °C. It could be shown that at temperatures 10 ± 2 °C, there was no peak signal. This could be due the fact that the optimal reaction temperature of the PP-L is usually around 37 °C. It could also be shown that at 35 ± 2 °C, the peaks decreased. This could be explained by the softening of the carbon paste. The best results were obtained at room temperature (22 ± 2 °C). Even though the ideal reaction temperature would be 37 °C, the good results enable the measurement at room temperature, which is an advantage when it comes to handling (see Supplementary Information Fig. S2).

Due to the fact that lipase reacts at the lipid-water interface, the ideal amount of substrate-paraffin oil solution was investigated. The substrate concentration was kept constant at 10 mM. Amounts of 50–200 μL lipid per measurement were tested. Volumes higher than 200 μL lipid caused a softening of the carbon paste, due to its lipophilicity. The volume of 100 μL lipid obtained the highest and most constant peaks. Therefore, the respective amount of substrate was dissolved in paraffin oil and 100 μL was injected into the measuring cell.

**Fig. 3:**
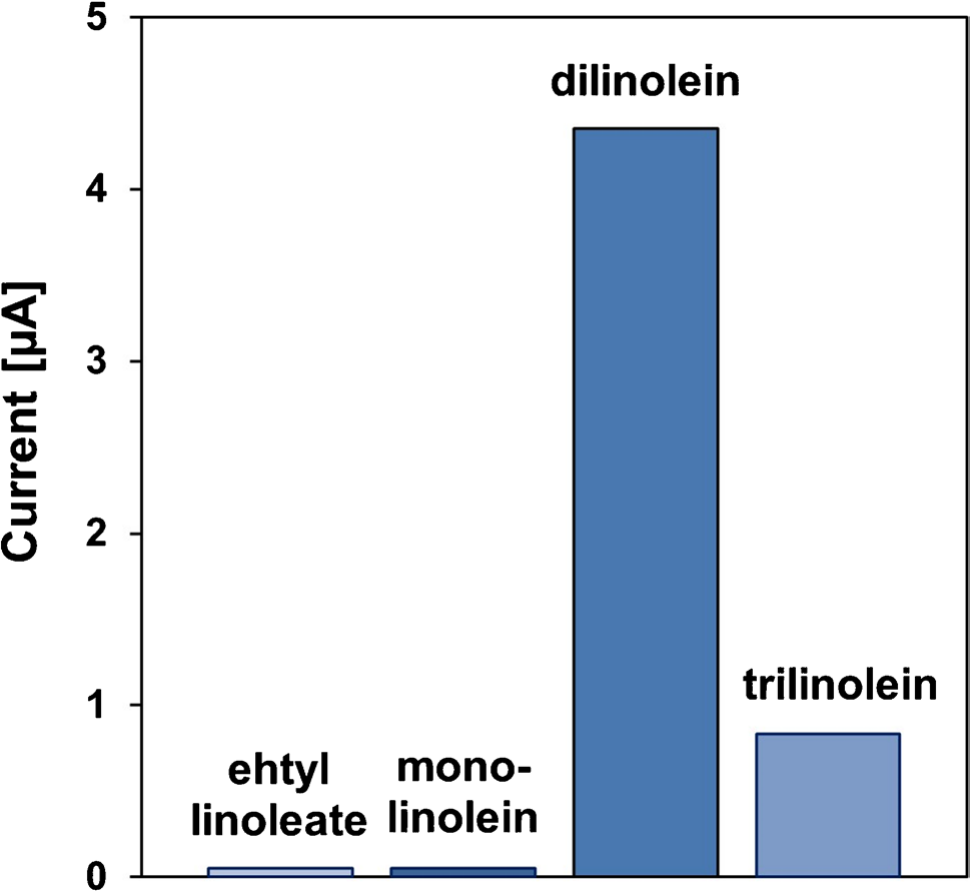
Substrate specificity investigations with ethyl linoleate, monolinolein, dilinolein, and trilinolein (each 0.2 µM) in sodium borate buffer (pH 9, 0.1 M) after 25-min reaction time

To additionally optimize the method and to investigate the substrate specificity, measurements with ethyl linoleate and three glycerol linoleates (mono-, di-, and trilinoleate) were carried out. No peaks were generated with ethyl linoleate, which confirms that PP-L most likely reacts with triglyceride structures. Due to the regiospecificity to the ester bond at the Sn-1,3 position of the triglyceride, the best results could be achieved with dilinolein, a 1,3-triglyceride (see Fig. 3). Hence, it was used as a standard substrate for the development of the activity assay.

Furthermore, the preparation of the PP-L stock solution was optimized. After various trials of centrifugation and filtration, it was determined that the best results were achieved by centrifuging the PP-LS for 2 min at 4 °C at 3000 rpm. For the electrochemical measurements, only the supernatant was used.

Since the investigations were carried out in weak alkaline medium, the hydrolysis can happen also without lipase. To investigate if the products were formed via enzymatic reaction, an inhibitor (Orlistat) with a high concentration (0.12 mg mL^−1^) was added (10 μL) to the PP-L containing measuring solution resulting in a total inhibition as no peak at the potential of + 0.35 V could be detected. Hence, the enzymatic reaction could be confirmed.

Finally, the optimized conditions were determined as follows: The measuring cell was filled with sodium borate buffer (0.1 M) at pH 9. Ten milligrams of PP-L was diluted in 1 mL water and centrifuged at 4 °C for 2 min with 3000 rpm, and 900 μL of the supernatant was injected in the measuring cell, followed by 100 μL substrate solution (10 mM). The measurement was carried out at room temperature.

### Determination of enzyme activity

In this work, the activity was defined as follows: 1 unit is the amount of enzyme that hyrolyzes 1 microequivalent substrate (= dilinolein) per minute at pH 9 at room temperature.

**Fig. 4:**
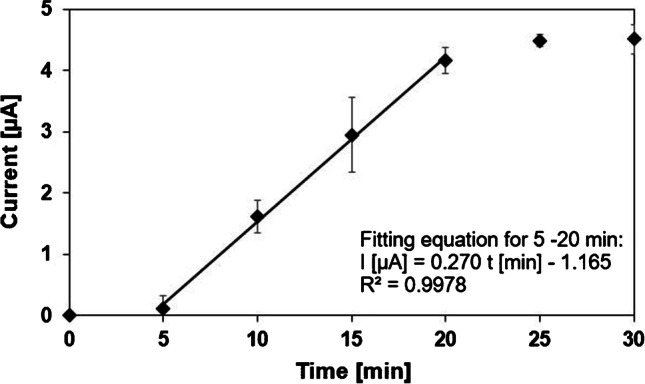
Time dependence of the current for the PP-L (200 U L^−^^1^) in the presence of dilinolein (10 mM) in sodium borate buffer (pH 9, 0.1 M); data was fitted between 5 and 20 min

The activity was calculated via the time dependency curve. For the calculation, the linear range between 5 and 20 min of the time curve was used (see Fig. 4). It could be determined that the peak increased by 0.270 μA per min.

With the help of the calibration function (*I* [μA] = 0.016 c [μg mL^−1^] − 0.6; *R*^2^ = 0.981) of linoleic acid, it could be observed that 54.4 μg mL^−1^ linoleic acid is formed per minute, which corresponds to 0.19 μmol mL^−1^ per min. The used PP-L powder contained 40% protein. This results in 0.13 units per mg PP-L powder corresponding to 0.32 units per mg of the protein in the powder.

### Analytical parameters

The validation of the PP-L activity assay was carried out by using dilinolein as standard substrate and a reaction time of 25 min. Linearity could be shown 20–300 U L^−1^ (per min) PP-L with a regression equation of *I* [μA] = 0.0285 activity [U L^−1^] + 0.677 and a correlation coefficient of *R*^2^ = 0.9992. The limit of detection (LOD) was determined to be 7 U L^−1^ and the limit of quantification (LOQ) is approximately 20 U L^−1^. LOD and LOQ were calculated by 3 and 10 SD, respectively.

**Fig. 5:**
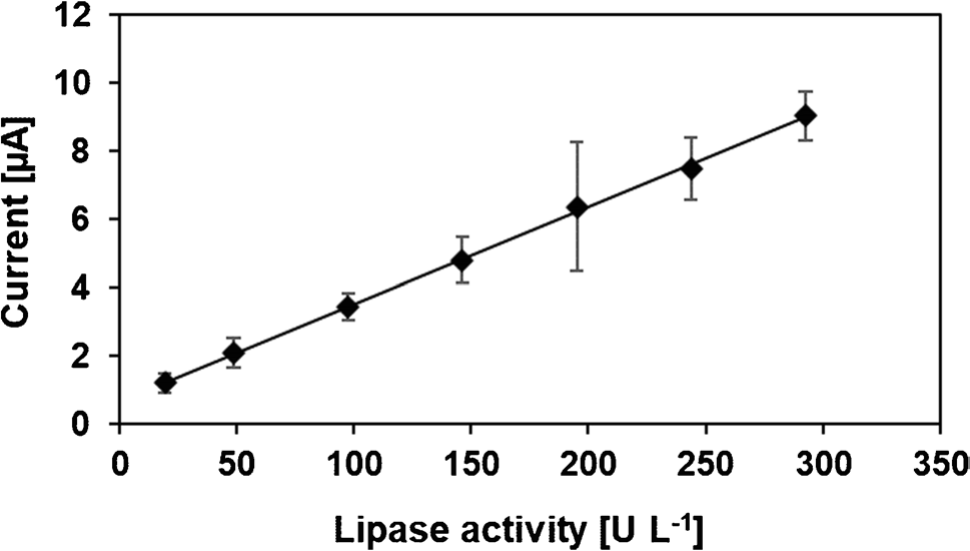
Calibration curve of optimized PP-L activity sensor with dilinolein as standard substrate (10 mM) after 25 min in sodium borate buffer (pH 9, 0.1 M); the peak at + 0.4 V was evaluated

The relative standard deviation (RSD) was found to be 11% (*n* = 6 different electrodes). RSD represents the reproducibility of the method. All analytical parameters are summed up in Table 1. The calibration curve of the optimized sensor can be seen in Fig. 5.

**Table 1:** Summary of analytical parameters (*n* = 6 different electrodes)

Parameter	Result
Linearity	20–300 U L^−^^1^ (per min)
LOD	7 U L^−^^1^
LOQ	20 U L^−^^1^
RSD^a^	11%

^a^Lipase activity = 100 U L^−1^

In order to determine the accuracy of the electrochemical assay, activity of untreated and treated (e.g., centrifugation or filtration) PP-L was determined and compared with the results achieved by titrimetric method. The results of these investigations are given in Table 2. Due to the comparable percentage decrease of the lipase activity, the accuracy of the elctrochemical assay could be confirmed.

**Table 2:** Comparison voltammetric method and titration method

Treatment of lipase	Decrease of activity [%] Voltammetric method	Decrease of activity [%] Titration method
Filtrated lipase (cellulose filter, 8–12 μm pore size)	84 ± 14	83 ± 1.6
0.15 μg mL^−^^1^ Orlistat	43 ± 1.3	42 ± 0.5

When compared to other already existing methods, it can be said that the developed voltammetric activity assay can be performed with a stable and cheap substrate, as well as a wide linear range and a low LOD (see Table 3). Furthermore, it can be used at room temperature and without pre-treatment of the sample or the electrode.

**Table 3:** Comparison of existing methods for lipase activity determination

Method	Substrate	Linear range	LOD	Lipase source	Reference
Electrochemical method	9-(5′-Ferrocenylpentanoyloxy)-nonyl disulfide (FPONDS)	4–400 U L (glycerol tributyrate)	n.d	*Thermomyces lanuginosus*	[11]
FIA with electrochemical detection	Olive oil	10–1500 U L^−^^1^	10 U L^−^^1^	Porcine pancreatic lipase	[4]
Voltammetric method	p-Nitrophenyl acetate	10–70 ppb (methyl parathion)	87.72 ppb	*Candida rugosa*	[13]
Voltammetric method	Polyaniline	0 and 225 IU L^−^^1^	0 IU L^−^^1^	*Candida rugosa*	[14]
Voltammetric method	Glyceryl dilinoleate	20–300 U L^−^^1^ (per min)	7 U L^−^^1^	Porcine pancreatic lipase	This work

### Determination of kinetic parameters

**Fig. 6:**
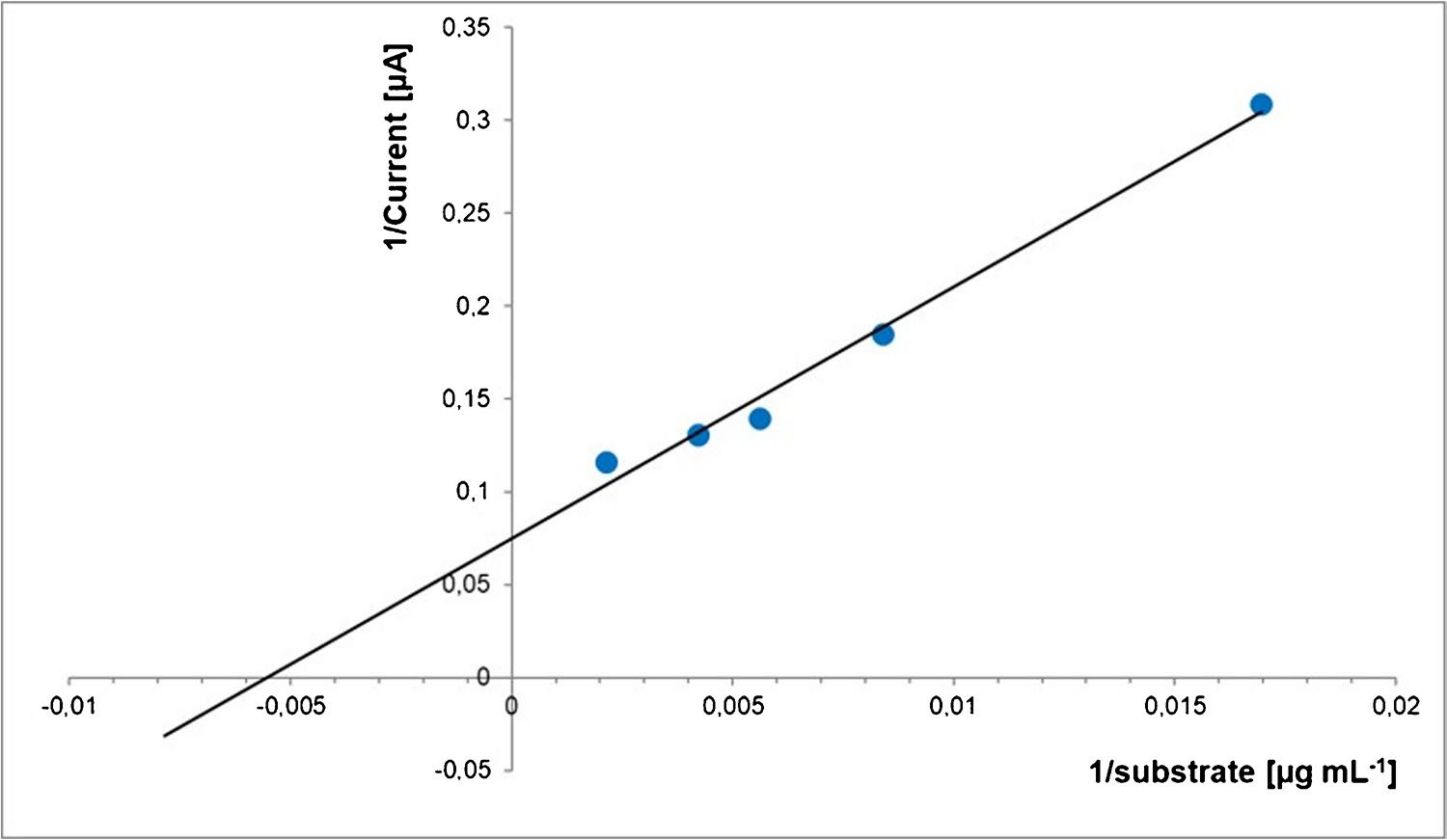
Plot of the inverse of the current after 25 min vs. the inverse of the dilinolein concentration (60, 120, 180, 240, and 470 µg mL^−1^) by using PP-L (200 U L^−^^1^) performed at the carbon paste electrode in sodium borate buffer (pH 9, 0.1 M) (*n* = 5)

The kinetic parameters of PP-L, the maximum-current (*I*_max_), and the Michaelis Constant (*K*_M_) were determined from the plot as about 13.4 μA and 182 μg mL^−1^ or 0.29 mM, respectively (see Fig. 6). The latter corresponds to current literature sources with *K*_M_ between 0.13 and 0.21 mM at pH 8 and 40 °C [22]. Evidently, the maximum velocity *V*_max_ cannot be determined directly, because currents are used in the model. Nevertheless, the currents should be directly proportional to the velocity.

### Application

**Fig. 7:**
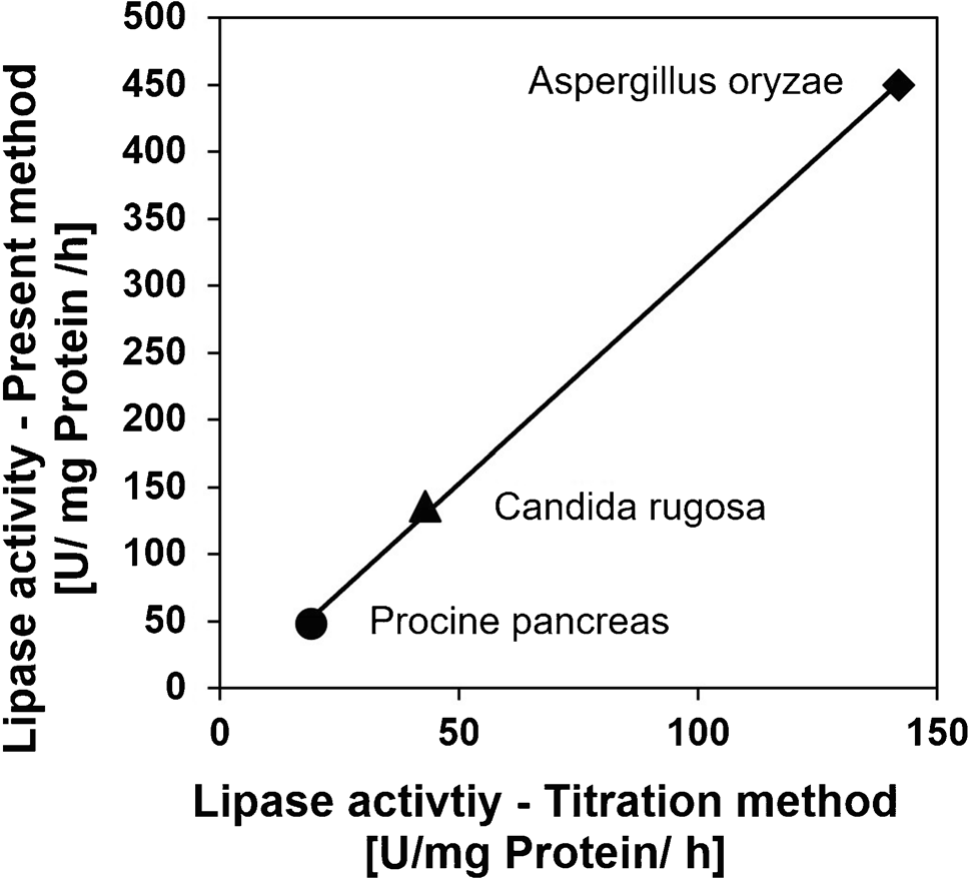
Correlation of the lipase activity of three different sources, obtained with the titration method with that of the developed method

To test the applicability of the developed method, two additional lipases were tested (lipases from *Aspergillus oryzae* and *Candida rugosa*), the protein content was determined via Biuret method and the obtained results were compared with the titration method. The found activities differ due to the different methods used (difference in temperature and pH). Furthermore, dilinolein as substrate is more representative for lipids and oils than triacetin. However, a correlation of the lipase activity detected via titration with the lipase activity obtained with the developed method can be seen in Fig. 7. The biggest advantage compared to the titration method is the higher reproducibility.

According to literature, less reproducible data might be obtained with the titration method due to the high amount of free fatty acids generated during the long incubation time of the titration and the inadequate buffering of said fatty acids [4]. This problem does not occur at the developed assay method, because the low amount of free fatty acids produced during the much shorter reaction time. Furthermore, the necessary sample amount is relatively small, compared to the titration method.

### Investigation of inhibitory effects

**Fig. 8:**
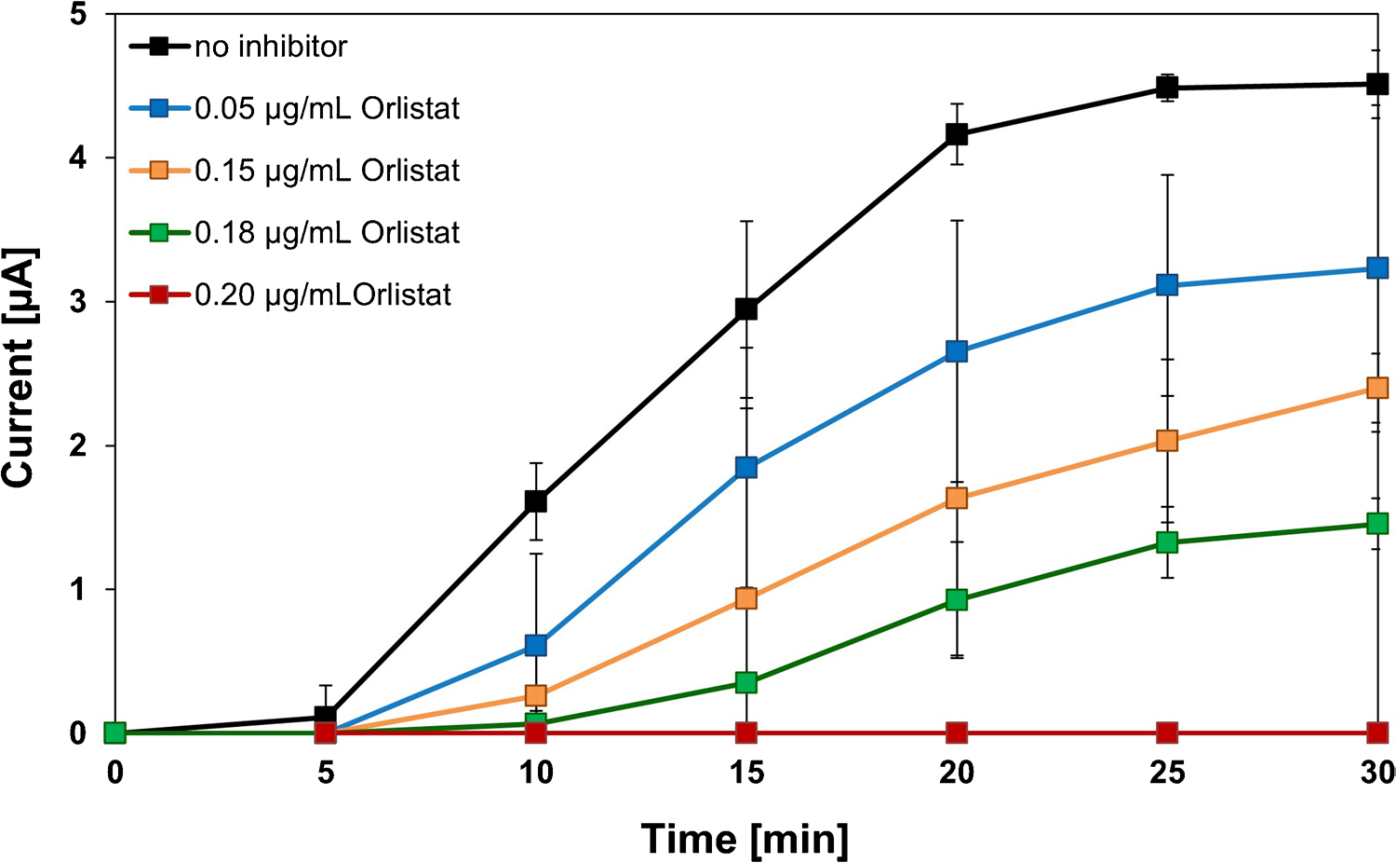
Time dependence of the current for the inhibition of Orlistat with concentrations of 0.05–0.20 µg mL^−1^ with PP-L (200 U L^−^^1^) and dilinolein (10 mM) in sodium borate buffer (pH 9, 0.1 M)

If the developed system can be used for characterizing the effect of inhibitors on lipases, time-dependent measurements with Orlistat were carried out. Orlistat is, as mentioned above, a specific and potent inhibitor of the pancreatic and gastric lipases. It forms a covalent bond with the active serine site of the lipase, thus inactivating the lipase and preventing it from hydrolyzing dietary fats. The undigested triglycerides are then eliminated by the fecal route. [16, 17, 23]. Therefore, various concentrations of the inhibitor in the range of 0.05–0.20 μg mL^−1^ were investigated. The respective amount of inhibitor was diluted in ethanol, and 10 μL was injected into the measuring cell. Then, the time-depending inhibition of the PP-L activity was measured. As expected, an inhibition occurred, with a 50% inhibition at a concentration of 0.15 μg mL^−1^ Orlistat and a total inhibition at 0.20 μg mL^−1^ (see Fig. 8). The ethanol amount was found to be neglectable.

**Fig. 9:**
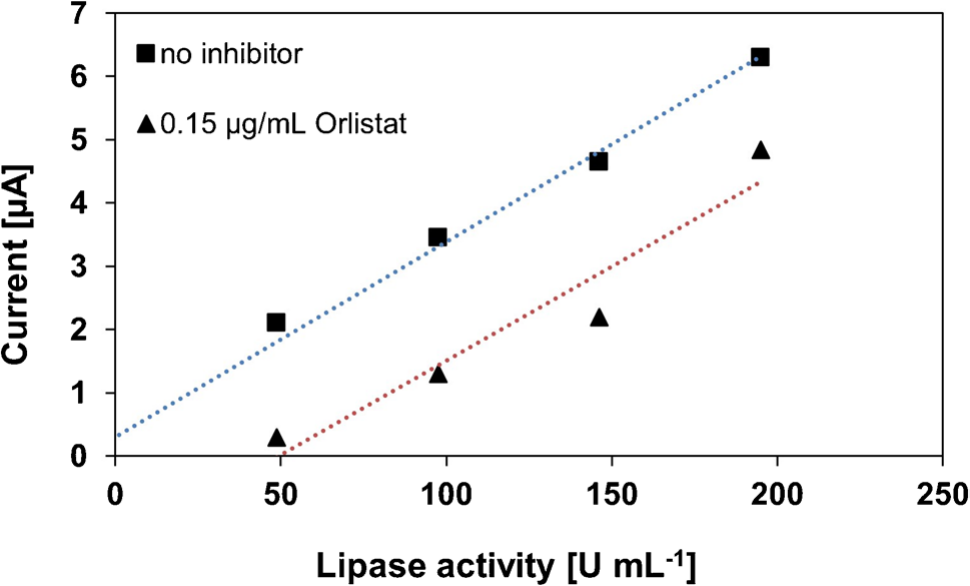
A plot of *I*_max_ versus PP-L activity with and without Orlistat in the presence of dilinolein (10 mM) after 25-min reaction time in sodium borate buffer (pH 9, 0.1 M)

According to literature [17, 24], Orlistat is a potent, irreversible inhibitor of the lipase. As it is not possible to distinguish between reversible (non-competitive) and irreversible inhibitors via Lineweaver–Burk plot, experiments were performed by measuring the dependence of the *V*_max_ (corresponding to *I*_max_) and the activity of the enzyme, whereby the substrate concentration was kept constant. An irreversible inhibitor would show the same slope as the non-inhibited lipase, whereas the non-competitive inhibitor would lead to a smaller slope, passing through the origin. In our case, the slopes *k* = 0.031 μA U^−1^ L without inhibition and *k* = 0.030 μA U^−1^ L with inhibition are practically the same, proving an irreversible inhibition of Orlistat (see Fig. 9).

## Conclusion

An electrochemical assay for the determination of lipase activity was developed. The assay offers a simple handling and a wide linear range at very low activity with a good precision. It uses only low-cost ingredients for the carbon paste and the sample preparation. The samples are only diluted with ultrapure water and centrifugated, using only the supernatant. No further pre-cleaning steps are necessary. It is therefore a simple and time-saving method for the determination of lipase activity. Furthermore, it can be used at room temperature and with a well-defined substrate, which is a great advantage for enzyme determination. No stabilizers are necessary, and the results are comparable with literature sources. The applicability of the developed method was successfully tested with two additional lipase sources. When compared to conventional methods, such as titration, the main advantage is the good reproducibility and the small sample amount needed. Furthermore, the assay can be also used for turbid or colored solutions. The sensor is also applicable for the characterization of enzyme inhibitors and screening of inhibitory effects.

## Supplementary Information

Below is the link to the electronic supplementary material.

Supplementary file1 (PDF 154 KB)
