# Supplementary material for: Voltammetric lipase activity assay based on dilinolein and a modified carbon paste electrode
Source: Anal Bioanal Chem. 2022 May 31;414(17):5033–41. doi: 10.1007/s00216-022-04135-y (PMC9234029; doi:10.1007/s00216-022-04135-y)
Supplement: Supplementary file 1 — Supplementary file1 (PDF 154 KB) [file 216_2022_4135_MOESM1_ESM.pdf]

Voltammetric lipase activity assay based on dilinolein and a modified carbon paste electrode

Anita Rogala [a], Julia Rechberger [a], Vanessa Vasold [a], Anchalee Samphao[b], Kurt Kalcher [c], Astrid Ortner [a] *

[a] Institute of Pharmaceutical Sciences, Department of Pharmaceutical Chemistry, University of Graz, Schubertstraße 1, 8010 Graz, Austria

[b] Department of Chemistry, Faculty of Science, Ubon Ratchathani University, Ubon Ratchathani 34190, Thailand

[c] Institute of Chemistry, Department of Analytical Chemistry, University of Graz, Universitätsplatz 1, 8010 Graz

email: astrid.ortner@uni-graz.at, phone: 0043/316 380 5372

*corresponding author

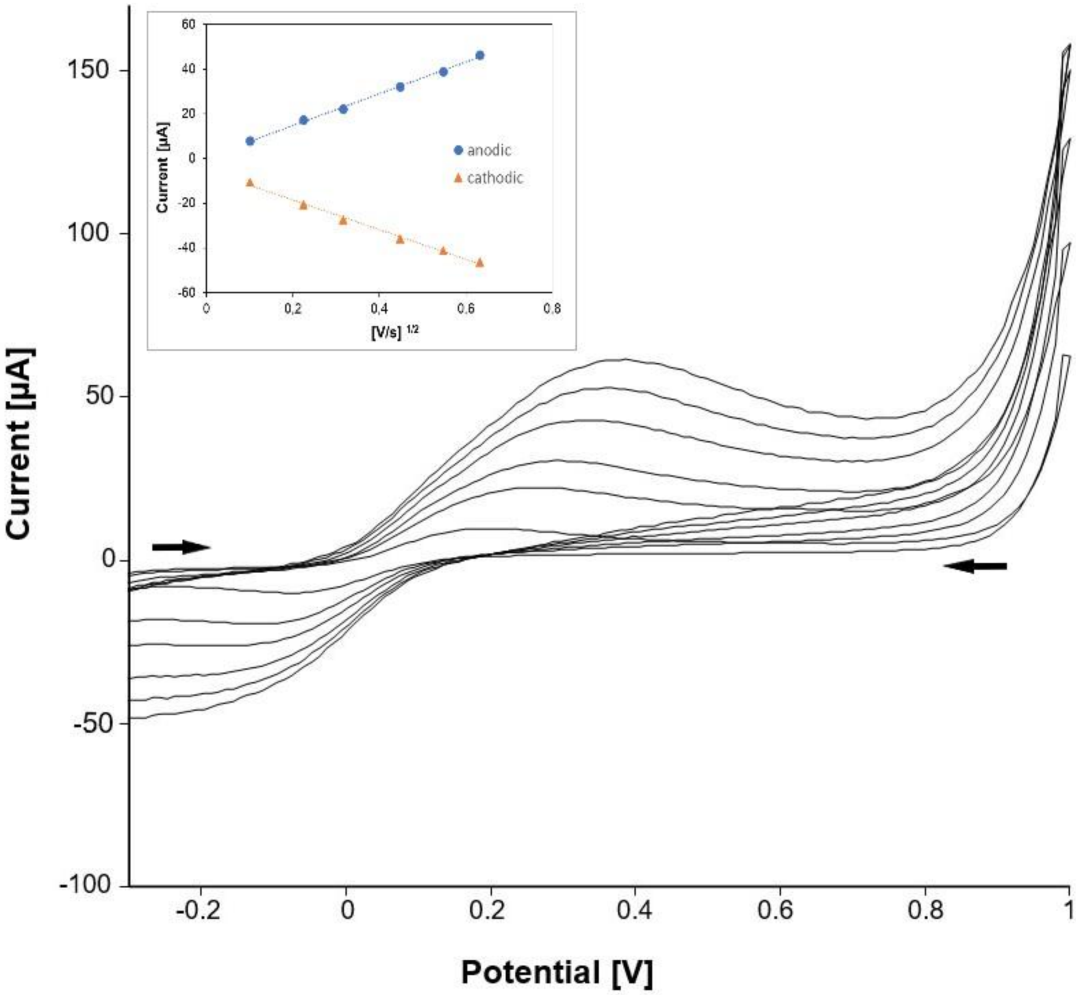

Fig. S1 Cyclic voltammetric measurements (CVs) with hexacyanoferrate in sodium borate buffer (pH 9, 0.1 M) using different scan rates (0.01 - 0.4 V s^{-1}) and showing a linear dependence of the peak current and the square root of the scan rate

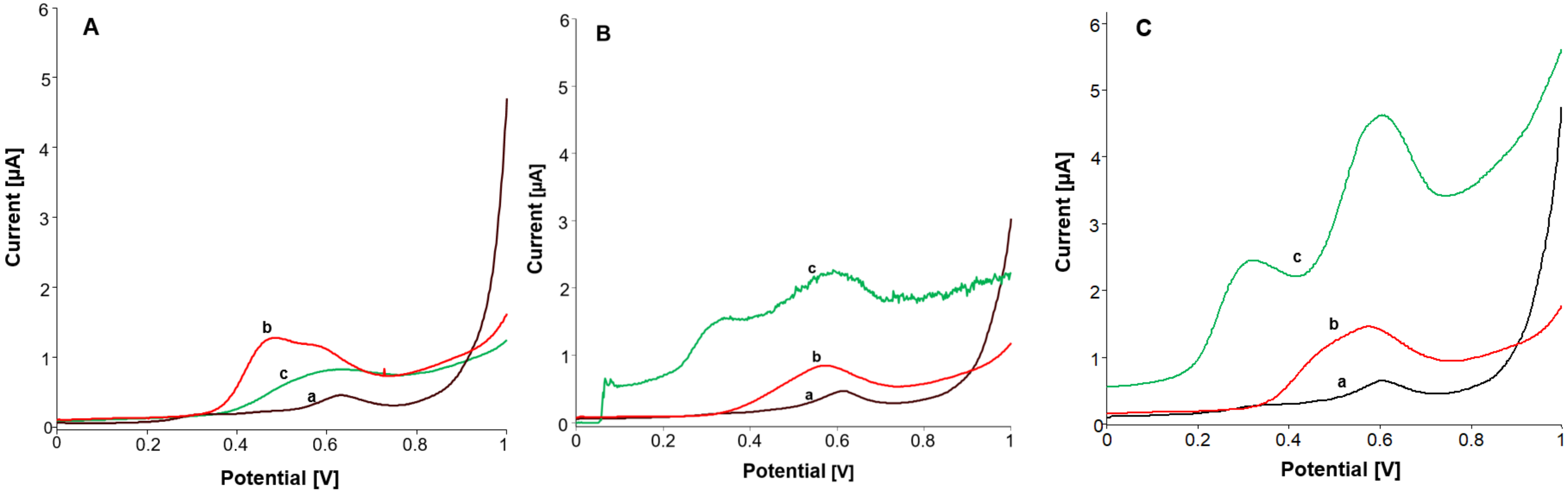

Fig. S2 Temperature dependence of lipase activity: blank (a), after addition of 900 µL PP-LS (10 mg mL^{-1}) (b), immediately after injection of 100 µL trilinolein (10 mM) (c) after 30 min reaction time at (A) 10 ± 2 °C, (B) 35 ± 2 °C and (C) room temperature (22 ± 2 °C)
